# Biomechanical implications of walking with indigenous footwear

**DOI:** 10.1002/ajpa.23169

**Published:** 2017-01-19

**Authors:** Catherine Willems, Gaetane Stassijns, Wim Cornelis, Kristiaan D'Août

**Affiliations:** ^1^ Department of DesignKASK, School of ArtsGent9000Belgium; ^2^ Department of Physical Medicine and RehabilitationUniversity Hospital Antwerp University of AntwerpEdegem2650Belgium; ^3^ Department of Soil ManagementGhent UniversityGent9000Belgium; ^4^ Department of Musculoskeletal BiologyInstitute of Ageing and Chronic Disease University of LiverpoolLiverpoolL7 8TXUK; ^5^ Department of BiologyUniversity of AntwerpAntwerp2020Belgium

**Keywords:** footwear, gait, impact, indigenous, kinematics

## Abstract

**Objectives:**

This study investigates biomechanical implications of walking with indigenous “Kolhapuri” footwear compared to barefoot walking among a population of South Indians.

**Materials and methods:**

Ten healthy adults from South India walked barefoot and indigenously shod at voluntary speed on an artificial substrate. The experiment was repeated outside, on a natural substrate. Data were collected from (1) a heel‐mounted 3D‐accelerometer recording peak impact at heel contact, (2) an ankle‐mounted 3D‐goniometer (plantar/dorsiflexion and inversion/eversion), and (3) sEMG electrodes at the m. tibialis anterior and the m. gastrocnemius medialis.

**Results:**

Data show that the effect of indigenous footwear on the measured variables, compared to barefoot walking, is relatively small and consistent between substrates (even though subjects walked faster on the natural substrate). Walking barefoot, compared to shod walking yields higher impact accelerations, but the differences are small and only significant for the artificial substrate. The main rotations of the ankle joint are mostly similar between conditions. Only the shod condition shows a faster ankle rotation over the rapid eversion motion on the natural substrate. Maximal dorsiflexion in late stance differs between the footwear conditions on an artificial substrate, with the shod condition involving a less dorsiflexed ankle, and the plantar flexion at toe‐off is more extreme when shod. Overall the activity pattern of the external foot muscles is similar.

**Discussion:**

The indigenous footwear studied (Kolhapuri) seems to alter foot biomechanics only in a subtle way. While offering some degree of protection, walking in this type of footwear resembles barefoot gait and this type of indigenous footwear might be considered “minimal”.

## 1 Introduction

Locomotion is crucial for humans, which are unique in having evolved into striding bipeds with an efficient walking gait. Walking and running ultimately boils down to the mechanical challenge of generating an impulse by means of the interaction between feet and the ground. During most of human history this foot‐ground interaction has involved a bare foot interacting with a natural (but highly variable) substrate. Only very late in their evolution, long after they had become anatomically modern (D'Août, Pataky, De Clercq, & Aerts, 2009; Kuttruff, Dehart, & O'brien, 1998; Trinkaus & Shang, 2008) humans became habitually shod.

Archaeological evidence suggests that footwear was probably invented in the middle upper Palaeolithic, ca. 25 thousand years ago (Trinkaus, 2005). Throughout most of its history, however, indigenous footwear probably remained very basic and was made from plant fibres or a simple leather construction as seen, for instance, in ancient Egyptian (Veldmeijer, 2009a, 2009b, 2009c; Veldmeijer & Clapham, 2011) and Roman (Sesana, 2005) footwear. The daily use of constricting footwear, with features such as a firm heel cup, arch support, cushioning, and motion control, is a recent phenomenon. In running shoes, for example, development of such features mostly occurred since the 1970s (Lieberman et al., 2010; Shorten, 2000), although interest in barefoot running and in various types of more or less “minimal” shoes has increased during the last decade. For comprehensive overviews of the latter, see dedicated volumes of Footwear Science vol. 5(1), 2013 and the Journal of Sport and Health Science vol. 3, 2014. We note that various types of highly decorated footwear have long existed for cultural purposes (Alton, Baldey, Caplan, & Morrissey, 1998; Riello & McNeil, 2006), but these were not owned or worn on a daily basis by a large part of the population.

Footwear interacts at the foot‐ground interface and can thus be expected to have a major influence on the mechanics of gait. A large body of work has focused on this influence for running (e.g., Altman & Davis, 2012; Kelly, Lichtwark, Farris, & Greswell 2016; Lieberman, 2014; Lieberman et al., 2015 and references therein), but the effects on normal walking in healthy subjects have received relatively little attention.

With regard to footwear, it has been suggested that habitual use of footwear can cause pathological changes (Fong Yan, Sinclair, Hiller, Wegener, & Smith, 2013; Hoffmann, 1905; Zipfel & Berger, 2007) and that (in native populations) a habitually unshod foot is healthier than a habitually shod foot (Mafart, 2007; Zipfel & Berger, 2007). Causal relationships are difficult to demonstrate, but Sachitanandam and Joseph (1995) show, for instance, that adults who began to wear closed toe‐shoes before the age of six had a higher prevalence of flat feet compared to those who began wearing shoes only after the age of six. It has also been found that shoes can restrict the natural motion of the bare foot and impose a specific foot motion pattern on individuals during the push‐off phase (Morio, Lake, Gueguen, Rao, & Baly, 2009).

Extensive research of running barefoot or in minimal footwear has revealed relationships between changes in footwear and changes in strike pattern (Bonacci et al., 2013; Daoud et al., 2012; Kerrigan et al., 2009; Lieberman et al., 2010; Lieberman, 2012a, 2012b; Perl, Daoud, & Lieberman, 2012). The advantages of barefoot running, such as lower injury rate, are still debated (Daoud et al., 2012; Hatala, Dingwall, Wunderlich, & Richmond, 2013b; Jenkins & Cauthon, 2011; Lieberman et al., 2010), but it has been shown that barefoot running with a forefoot strike involves a lower impact peak than shod running with a heel strike. Such a relationship is not to be expected for walking, however, since in healthy subjects walking always involves a heel strike, and (all else being equal) we would expect a higher impact when barefoot than when shod.

In this article, we explore the effect of footwear on human walking by studying a South Indian population that (a) is used to barefoot as well as (b) shod walking, using basic indigenous footwear on a daily basis. The experiment was done on artificial (man‐made, paved) substrates. In addition, we have repeated the same test on a natural substrate. The focus of this paper is entirely on between‐footwear conditions, using two substrates as separate cases for validation.

We compare walking barefoot versus walking with indigenous footwear with a focus on kinematics, kinetics (accelerometry), and muscle activity. More specifically, we will compare (1) the peak acceleration of the foot at initial impact, (2) the main rotations of the ankle joint, plantarflexion/dorsiflexion, and inversion/eversion, and (3) the activity patterns and magnitude of two major external foot muscles, that is, the m. tibialis anterior (a dorsiflexor) and the m. gastrocnemius (a plantar flexor).

We test the hypotheses that walking barefoot, compared to shod walking, involves higher impact accelerations, slower ankle rotations over a larger range, and a higher muscular activation.

## 2 Materials and methods

### 2.1 Subjects

Ten healthy adult volunteers were recruited from the local population in Athani, a small rural village in the state of Karnataka, South India (Table 1).

**Table 1:** Subject info

Population *n*	Age (years)/avg ± SD range	Mass (kg)/Avg ± SD range	Stature (m)/Avg ± SDr ange	BMI/Avg ± SD range
10 (*f* = 3, *m* = 7)	35.8 ± 8.8 22–53	53.6 ± 10.4 44–73	1.60 ± 0.09 1.45–1.75	20.7 ± 3.1 16.4–25.9

All subjects were habitually Kolhapuri (indigenous footwear) wearing adults and walked barefoot during childhood up to approximately age six. For details about this specific footwear see Willems (2013). The subjects had no apparent foot or orthopaedic problems, and they had a normal gait. The subjects participated on a voluntary basis, were informed of the protocol by a local translator, and gave written informed consent according to the protocols approved by the ethical committee of the University of Antwerp. Prior to the recordings the subjects were weighed and measured and they answered a short questionnaire about footwear habits and recent injuries of feet and ankles.

### 2.2 Footwear

**Figure 1:**
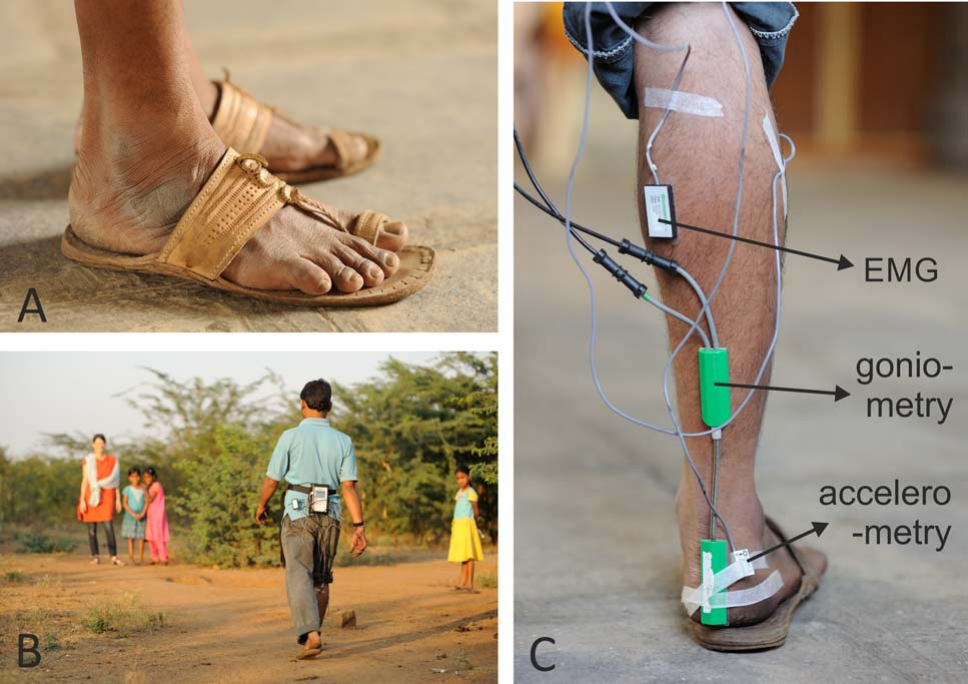
(A) Kolhapuri footwear, (B) Subject in the field (natural substrate), (C) Subject on the artificial substrate

Kolhapuri footwear, a type of sandal made entirely from buffalo skin, originates from the districts of Karnataka and Maharashtra and is commonly used all over India. The sandal (Figure 1A)—or chappal, as it is locally called—is made out of bag tanned buffalo leather, using babul bark and myrobalan fruits. All parts of the chappal—sole, uppers, and heel—are from this leather. The sandal is characterized by a toe ring and an instep band. Often a toe strap, woven in leather, is attached passing from the instep band to a point adjacent to toe ring on the sole. The instep band is fixed between out‐ and insole and the toe loop into a slot near the toe. The whole sandal is stitched with a leather rope, taken from the tail portion of the same bag tanned leather. The sole stitching is all around the sole, and no glues are used. The footwear does not constrict the feet, has no extra arch support and a very low heel rise (a few mm).

We measured thickness of the Kolhapuri sandals as worn by our subjects at four locations using callipers (all values are presented as the average ± standard deviation). The medial midfoot region is least prone to wear and, therefore, its thickness reflects the raw material thickness best: it was 9.76 ± 2.86 mm. The heel is more prone to wear and one or more extra layers of leather are usually added: heel thickness was 14.95 ± 6.35 mm. Under the hallux and the metatarsal region, no extra layer of leather is added but there can be substantial wear of the material; thickness was 7.81 ± 2.73 mm and 7.90 ± 2.49 mm, respectively.

The thickness of vegetable tanned buffalo leather is about 3 mm and the density is about 0,640 g/cm^3^, which is substantially less than that of natural rubber (about 0.930 g/cm^3^). The Kolhapuri footwear used in this study has an average mass of approximately 100 g for European size 37 and approximately 150 g for European size 42.

### 2.3 Substrate and footwear mechanical properties

Subjects were tested on large flat tiles of hard stone, this was considered as extremely stiff for the purpose of this paper (Figure 1A,C). In addition, and for reasons of comparison, we repeated the same test on a second substrate, which was a natural substrate in the outskirts of the village (Figure 1B). To characterise its mechanical properties, undisturbed 100 cm^3^ samples of the natural substrate (soil) were taken (we used standard 5 cm deep cores) from the site of the outside recordings during the time of the gait analysis in January 2010. In Athani, the winter is usually dry with temperatures around 20°C. Particle‐size analysis with the pipette method of Gee and Bauder (1986), showed that the natural substrate had a clay loam texture according to the USDA classification (Soil Survey Staff, 1999). The samples were also subjected to a compression test in which the resistance to compression was measured with depth, i.e., as the soil deformed. This was done with a laboratory type T‐5001 penetrometer (JJ Loyd Instruments Ltd., Southampton, UK) on the undisturbed samples using a metal plate with a surface area equal to that of the samples (∼20 cm^2^). Prior to the tests, the samples were brought to a field capacity moisture condition (i.e., soil moisture at 33 kPa matric suction), which corresponds to a situation after which water has been drained by gravity from the natural substrate (soil) (Hillel, 1998). A 50 N load cell was used to drive the metal plate onto the sample at a constant speed of 2 mm min^−1^.

**Figure 2:**
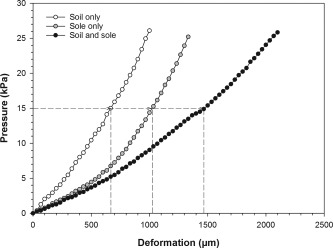
Stress‐deformation curves for indentation of samples using a 19.64 cm^2^ round stud. *x* = deformation (μm); *y* = pressure (kPa). Regression equations (2^nd^ order polynomial): for the soil + sole sample*y* = 10.1219e − 6 x^2^ + 0.0155911482 *x*; for the soil only sample *y* = 2.9631e − 6 *x* ^2^ + 0.0060091733 *x*; for the sole only sample *y* = 11.8267e − 6 *x* ^2^ + 0.0026848563 *x*. Soil thickness, 50 mm; sole thickness, approximately 6 mm (i.e., two layers of buffalo leather). The slope of the curves is a measure for stiffness of the samples. Note that the soil alone is stiffer than the sole alone. The soil + sole has the lowest stiffness. The dashed lines provide an example of results at a stress of 15 kPa. At this pressure, the soil alone yields approximately 650 μm, the sole alone yields approximately 1000 μm and a combined sample yields approximately 1450 μm. Please note that the sum of soil and sole deformation is not an exact mathematical match for the combined deformation, as the three curves result from different experiments, with slight sample variation

The measured resistance is a measure of the natural substrate's stiffness. Similar tests were performed on a section of leather used to manufacture the sole of Kolhapuri footwear and on a stacked sole placed on top of the soil sample. Results are shown in Figure 2.

The test continued until a maximum resistance was reached (∼24 kPa) with the used load cell. While realizing that the materials are not linearly elastic, we estimate from the data in Figure 2 (white circles) that the Young's modulus (a measure for elasticity) of the natural substrate is ∼1.15 MPa. No measurements were done with the stone tiles, since they would not be subjected to compression with the load cell used. For example, granite (the presumed material of the artificial substrate in this paper) has a typical Young's modulus of approximately 50 GPa and even soft limestone has a Young's modulus of at least several GPa (see, for instance, engineeringtoolbox.com), this implies that the relative deformation of these materials is at least three orders of magnitude smaller than the materials considered in Figure 2.

We note that these results should be treated with caution and are only intended to give an indication of substrate and footwear stiffness. Whereas the sole thickness used was the same as that used in shoe manufacturing, we used a single core type for soil characterization. Cores with different dimensions could yield slightly different compression in such tests. With this caveat in mind, the results show that the soil is stiffer (but of the same order of magnitude) than the leather sole. Thus, the natural substrate representative for this South Indian region is not considered soft.

### 2.4 Instrumentation and data collection

Prior to the experiments, subjects were instrumented as follows (Figure 1C). A 3D accelerometer (Biometrics ACL300) was fitted to the skin on the lateral side of the right calcaneus with double side tape and strapped tightly with strong medically approved tape. A twin axis goniometer (Biometrics SG) was fitted to the skin at the level of the right tuber calcanei, so that one axis measured the simple rotations dorsiflexion/plantarflexion (i.e., rotations in the sagittal plane) and the other axis measured inversion/eversion (i.e., rotations in the frontal plane). In this paper, pronation is a complex motion that involves dorsiflexion and eversion, whereas supination involves plantarflexion and inversion. Two surface‐electromyographic (sEMG) electrodes (Biometrics SX230) were attached to the skin overlying the right m. tibialis anterior and the m. gastrocnemius medialis. As an ankle extensor, the m. gastrocnemius produces 80–95% of net energy during walking (see Winter, 1983, 1991) and is thus a proxy for overall muscular activity in the leg during walking. The m. tibialis anterior is an antagonistic muscle to the m. gastrocnemius and acts as an ankle flexor, or eccentrically as a shock absorber during the initial heel strike when the ground reaction force produces an ankle extension moment. We therefore deemed this muscle to be important in the current study which addresses different footwear and substrates, potentially influencing the mentioned ground reaction force. Both muscles are easily accessible for surface‐mounted EMG electrodes. A neutral electrode was worn on the wrist.

All data were stored as text files on a belt‐mounted Biometrics DataLog unit and transferred to a PC for analysis after the full set of experiments. Data acquisition rate was 1000 Hz for the EMG and accelerometry. LaFortune and Hennig (1992) sampled accelerometry at 1000 Hz and found that 99% of the signal was below 60 Hz. Goniometry was sampled at 100 Hz.

During the experiments (January 2010), the subjects walked at a self‐selected, voluntary speed after several habituation trials. The conditions were: Barefoot on the Artificial substrate (BA) and Shod on the Artificial substrate (SA). In addition we measured: Barefoot on the Natural substrate (BN) and Shod on the Natural substrate (SN). All equipment remained on the subject throughout the experiment, which was possible because the footwear considered here has no heel strap. For each condition, subjects walked back and forth several times over a distance of approximately 10–15 m. Strides for analysis were from the middle, steady‐state sections (discarding the initial and terminal three strides), yielding approximately 20 steps per subject per condition. The complete data set consists of nearly 800 steps for which all data types are available, except for the sEMG data of subject 10 because of a technical failure.

Lateral‐view whole‐body video recordings were made at 50 fps for the trials on the artificial substrate using a full‐HD video recorder. Spatial calibration was performed using tape markers on the ground spaced at 1m intervals. In addition to the walking trials, a static standing trial was recorded for each subject.

In our first set of experiments we were unable to collect speed data during the experiments on the natural substrate. We have done a second field visit in which we collected speed data for the natural as well as the artificial substrate in July 2016.

### 2.5 Analysis: impact and spatio‐temporal gait

Impact was assessed as the magnitude of the vector sum (that is, using the *x*, *y*, and *z* components) of the unfiltered acceleration peak at initial impact, to compensate for small differences in accelerometer positions between subjects.

**Figure 3:**
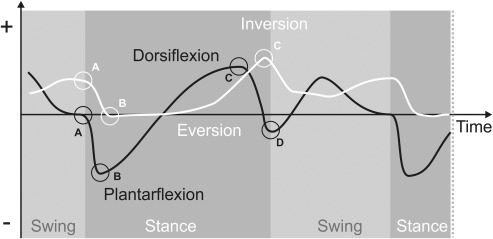
Ankle kinematics definitions. (A) Magnitude of plantar flexion/dorsiflexion and inversion/eversion at initial contact; time is defined as zero. (B) Minimal magnitude and timing of plantar flexion/dorsiflexion and inversion/eversion which occurs in early stance. (C) Maximal magnitude of plantar flexion/dorsiflexion and inversion/eversion which occurs in late stance. (D) Magnitude and timing of plantar flexion/dorsiflexion at toe‐off. See Figures 5 and 6 for results

Speed was measured for the trials on the artificial surface by dividing the distance covered in three full strides (as seen on the calibrated lateral‐view video recordings) by the corresponding duration. Stride duration was measured as the time between two consecutive strikes of the right foot using the accelerometer signal, as this yielded a consistent sharp peak at initial ground contact. Stance duration is the time from heel contact to toe off (i.e., the time from A to D on the plantarflexion‐dorsiflexion plot Figure 3). The duty factor is expressed as the percentage of time the foot is on the ground, that is, stance time/stride time (× 100%).

An Analysis of Variance (ANOVA) revealed that there was a significant interaction between the factors Subject and Condition when looking at speed (shod versus barefoot, both on the artificial substrate). The difference between footwear conditions was very small (shod: 1.27 ± 0.1 m/s; unshod: 1.30 ± 0.14 m/s) and not statistically significant. The speeds that we measured closely reflect the normal range of walking speeds and also closely match speeds for minimal energy expenditure (see e.g., (Zarrugh, Todd, & Ralston, 1974)) as well as a walking speed often imposed in controlled settings (e.g., Zhang, Paquette, & Zhang, 2013, 1.3 m/s).

To compare speeds between substrates we calculated speed by timing a known distance of ten individuals in the same site. All recruited subjects were from the original community, of which six original subjects and four new subjects that were matched (sex, age, BMI) to the missing original subjects. All subjects did five walking trials in all four conditions. The inside and outside sites are the same as during the first experiment, and the same accounts for the footwear worn. For every trial, speed was measured as the average speed over 5.0 m by timing the walks between landmarks on the ground. We tested differences between substrates separately for the barefoot and for the shod trials using one‐way ANOVAs.

### 2.6 Analysis: ankle kinematics

All ankle values are expressed relative to static standing to compensate for slightly different mounting of the goniometer between subjects. For statistical analysis, we selected several landmark points from the continuous angular measurements. Plantarflexion‐dorsiflexion values were measured at initial contact, at maximal plantarflexion (occurring in early stance), at maximal dorsiflexion (occurring in late stance), and at toe‐off. Ankle inversion‐eversion angles were measured at initial contact, at maximal eversion (occurring in early stance), and at maximal inversion (occurring in late stance). We also measured the duration of initial fast eversion following initial contact and the duration of the slow re‐inversion during stance.

### 2.7 Surface electromyography

Raw sEMG data (in arbitrary units) were high pass filtered (25 Hz, (Hof, Elzinga, Grimmius, & Halbertsma, 2002)) and rectified. Subsequently, the time series were normalised to one stride. Next, the curves of average magnitudes were normalised to their individual maximum enabling a comparison of the pattern (as opposed to magnitude) of muscle activation between conditions. Consequently, total sEMG of the medial head of the m. gastrocnemius (GM) and the m. tibialis anterior (TA) was calculated by numeric integration of the data to compare the total amount of muscle activation between conditions. In the latter case, the overall maximal values per muscle and per subject were determined (across all conditions) and used to normalise the corresponding individual recordings.

### 2.8 Statistical analysis

The extracted kinematic, acceleration, and EMG data per stride were used for analyses in a repeated measures design. We treated measures as independent, as they are from multiple gait trials interrupted by static standing and turning. The data were analysed with a one way repeated measures MANOVA for each dependent variable, and with Subject as a random factor. Significance was accepted for *p* < .05.

## 3 Results

### 3.1 Spatiotemporal gait

Barefoot walking on the artificial substrate involves a stride duration that is slightly shorter than for shod walking (albeit *p* = .044). On the natural substrate we note a shorter stance duration for barefoot walking (*p* = .042).

We tested the differences between substrates separately for the barefoot and for the shod trials. For the barefoot trials, average speed was 1.236 ± 0.102 m s^−1^ on the artificial substrate and 1.324 ± 0.102 m s^−1^ on the natural substrate (*p* = .000). For the shod trials, average speed was 1.213 ± 0.112 m s^−1^ on the artificial substrate and 1.325 ± 0.162 m s^−1^ on the natural substrate (*p* = .000). Speed differences between different footwear conditions on the same substrate were not significant.

### 3.2 Impact acceleration

**Figure 4:**
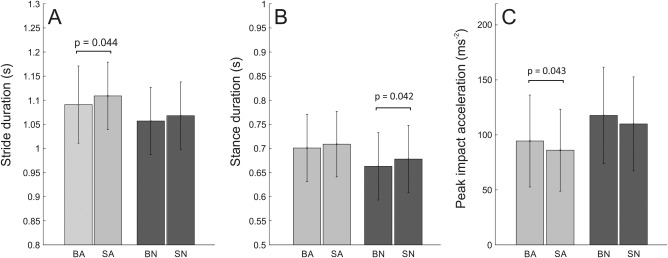
Spatiotemporal and impact results. From left to right: (A) stride duration, (B) stance duration, (C) peak impact acceleration which occurs at initial contact (m s^−2^). Abbreviations: BA, Barefoot Artificial & SA‐Shod Artificial; BN, Barefoot Natural & SN, Shod Natural

Initial impact is associated with relatively high and clearly recognisable accelerations across conditions. Difference in peak impact acceleration at initial contact, which in our experiments is always with the heel, are relatively small or absent and are only significant for the artificial substrate (Figure 4). The barefoot condition has higher impacts compared to shod walking (*p* = .043).

Peak impact differs most clearly between substrates, with the artificial substrate having lower values in both footwear conditions.

### 3.3 Ankle kinematics: plantar/dorsiflexion

The general pattern for ankle plantarflexion/dorsiflexion is followed. The foot lands at an almost neutral angle (compared to static standing) and then quickly plantarflexes by approximately 25–30°. This is followed by a slow dorsiflexion phase, when the body pivots over the stance foot and ends up being dorsiflexed by approximately 15°. In late stance, that is, during the push‐off phase, the ankle plantarflexes considerably to become plantarflexed by approximately 15–20° at toe‐off. During swing phase (not the focus of this paper) the ankle becomes more dorsiflexed again to allow for suitable toe clearance with the substrate (Figure 3).

**Figure 5:**
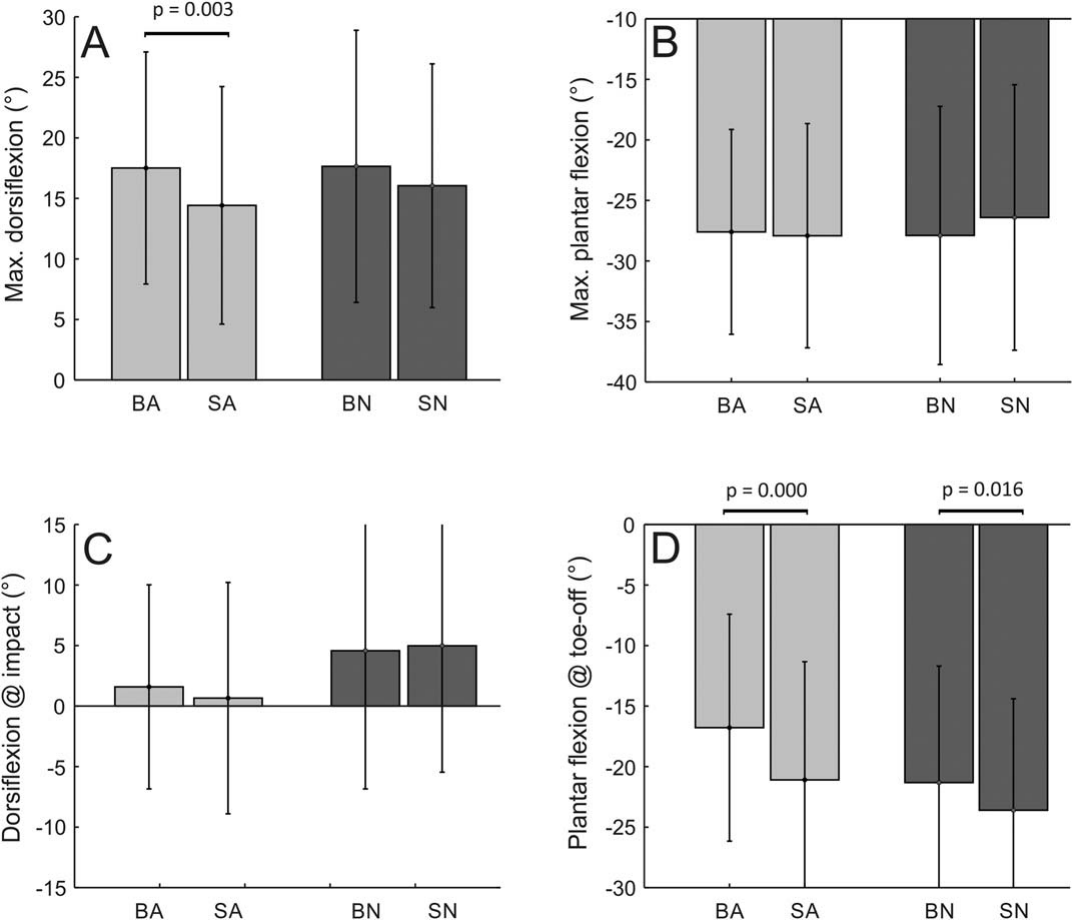
Plantarflexion‐dorsiflexion results (see Figure 3 for definitions). (A) maximal dorsiflexion (occurring in late stance), (B) maximal plantarflexion (occurring in early stance), (C) value at initial contact, (D) value at toe‐off. Note that all values are relative to static standing posture. Abbreviations: BA, Barefoot Artificial & SA‐Shod Artificial; BN, Barefoot Natural & SN, Shod Natural

Our quantitative analysis shows small differences between footwear conditions. Maximal dorsiflexion in late stance only differs between the footwear conditions on an artificial substrate, with the shod condition involving a less dorsiflexed ankle (Figure 5a). The barefoot condition shows a smaller degree of plantarflexion at toe‐off (Figure 5d).

Dorsiflexion at initial impact (Figure 5c) and maximal plantarflexion values in early stance do not differ between the footwear conditions (Figure 5b).

### 3.4 Ankle kinematics: eversion/inversion

The general pattern for ankle inversion/eversion (Figure 3) is the same for all conditions. At heel strike, the ankle is inverted by approximately 10° (relative to static standing). It then quickly everts to approximately neutral position, where it starts a slow re‐inversion peaking at approximately 20° near toe‐off.

**Figure 6:**
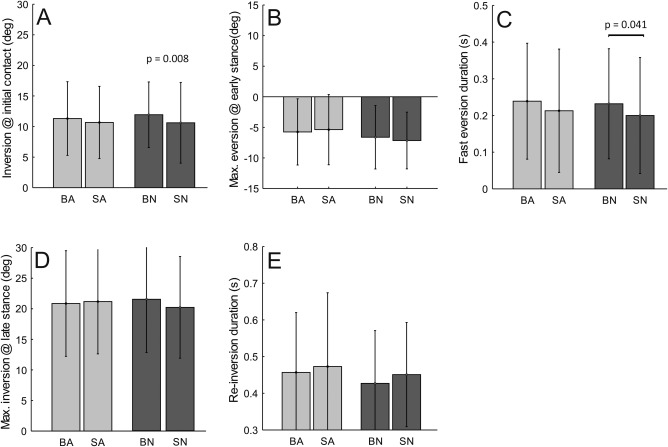
Ankle inversion‐eversion results (see Figure 3 for definitions). (A) value at initial contact, (B) maximal eversion (occurring in early stance), (C) duration of initial fast eversion following initial contact, (D) maximal inversion (occurring in late stance), (E) duration of the slow re‐inversion during stance. Note that all angle values are relative to static standing posture. Abbreviations: BA, Barefoot Artificial & SA‐Shod Artificial; BN, Barefoot Natural & SN, Shod Natural

Our quantitative analysis shows that ankle inversion at initial contact differs between barefoot and shod walking, being significant on the natural substrate: when barefoot, the ankle lands more inverted, by 1.3°, than when shod (Figure 6a). After initial contact, the ankle everts to a peak value. The results show similar values for both footwear conditions (Figure 6b). The duration of this rapid eversion motion shows a trend to be faster when shod on both substrates, but was only significantly different for the natural substrate (Figure 6c). The slow inversion motion is not significantly different for all conditions, both in terms of magnitude (Figure 6d) and in terms of duration (Figure 6e).

### 3.5 Electromyography

**Figure 7:**
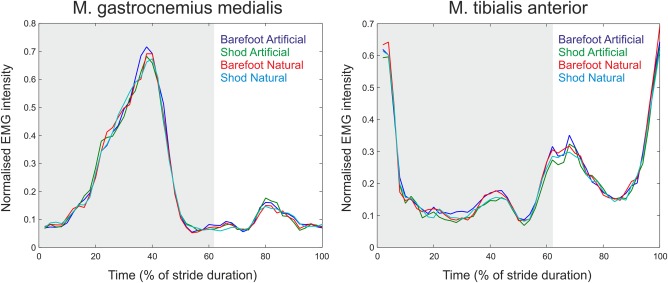
EMG profiles for the m. gastrocnemius medialis and for the m. tibialis anterior. All plots are averages for one stride, from right initial contact to the consecutive right initial contact, with all input data normalised to their individual maximal value. The grey area indicates stance phase. Note that shape of the normalised sEMG profiles is very similar between conditions

Patterns of sEMG activity are very similar between shod and barefoot conditions (Figure 7). The m. gastrocnemius medialis has one main activity peak, during the push‐off phase in mid/late stance, and a smaller peak just prior to touch‐down. The m. tibialis anterior shows a high activity around the instant of touch‐down, and a smaller peak during swing phase. Both patterns correspond well with those from the literature (e.g., Hof et al., 2002).

**Figure 8:**
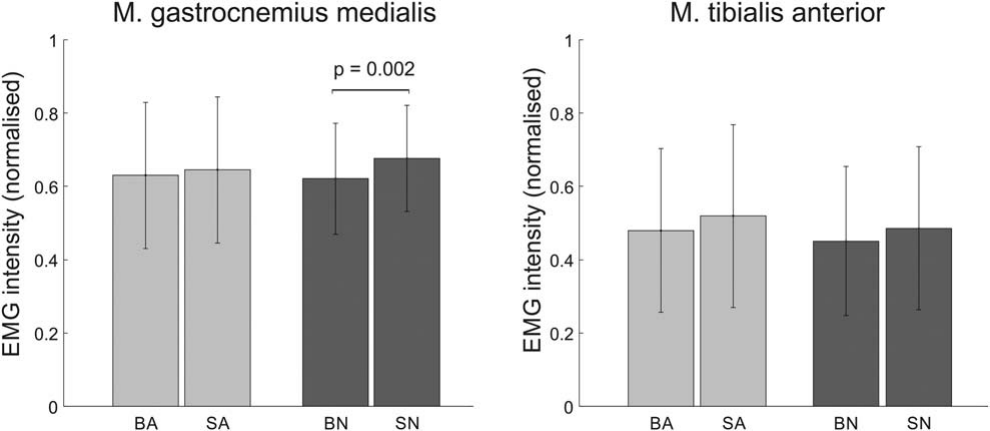
Results for total EMG during a stride. Data were normalised to the maximal value observed for the muscle in the individual subject. Abbreviations: BA‐Barefoot Artificial & SA‐Shod Artificial, BN‐Barefoot Natural & SN, Shod Natural

Total activity of the m. gastrocnemius medialis is similar in all conditions and we only found a difference on the natural substrate, where the barefoot condition involves lower muscle activation than the shod condition (Figure 8).

Total activity of the m. tibialis anterior does not differ between conditions but there is a trend for lower activity barefoot versus shod on the artificial substrate (*p* = .072, Figure 8).

## 4 Discussion

The study has highlighted differences and similarities in impact accelerations, ankle kinematics, and muscle activity of the m. tibialis anterior and the gastrocnemius while walking in different footwear conditions (that is, barefoot or indigenously shod). The focus of the experiment was on the artificial substrate, in addition we repeated the same test on a natural substrate.

With regard to our first hypothesis, we confirm that the barefoot condition yields higher impacts than the shod condition, but the differences are small and only significant for the artificial substrate. With regard to our second hypothesis, we note that the main rotations of the ankle joint are mostly similar between conditions barefoot and shod, with the exception of the rapid eversion motion that was faster when shod compared to barefoot, on the natural substrate. Finally, we reject our third hypothesis that the barefoot condition involves higher muscular activation.

Since we measured impact by means of accelerometry, and not force plates, we do not have data on ground reaction force. This was a deliberate choice, since the hard surface of the force plate differs substantially from a natural surface and is not suitable for measuring the effect of substrates. We measured soil density only during the dry season, which lasts an average of ten months a year. During the wet season the substrate might have different properties. It is expected that the differences between the artificial and the natural substrate during the wet season will be higher than in the present study.

As described above, walking speeds on the natural substrate were measured during a second experiment on the same substrate and with mainly the same subjects. The speeds on the artificial substrate are slightly lower than those measured previously during the first experiment which might be attributed to the partly different study population, the different method (video versus direct observation) or other factors. Since we only compare within‐subject in a single experiment this small difference is not of importance for our results.

Speed differences between footwear conditions on the same substrate were not significant. This is consistent with the findings of Price, Andrejevas, Findlow, Graham‐Smith, and Jones (2014), who found that walking speed on flip flops is comparable to barefoot walking. In general we noted a higher speed on the natural substrate for both footwear conditions. At the same time we saw that stride durations on the natural substrate are lower (i.e., higher stride frequency). In future studies it would be useful to collect speed data, e.g., by using high‐precision GPS data, for the very same trials as the kinematic and kinetic data.

Because of differences in methodology and protocol, the results obtained here cannot easily be compared with that of the other studies. Indeed, only a small number of papers compare barefoot and shod walking. We note that the outsole properties were similar for all subjects. We recognize that the relatively small sample size of this study may limit the generalizability of the results, and future research should include more subjects.

Previous biomechanical studies investigated the implications of walking in flip‐flops compared to barefoot and/or closed toe footwear (Chard, Greene, Hunt, Vanwanseele, & Smith, 2013; Morio et al., 2009; Shroyer & Weimar, 2010; Zhang et al., 2013) further support the hypothesis that all shoes, even the open‐toe footwear, yield different ankle kinematics compared to the barefoot condition. In line with Morio et al.(2009) our results show that the rapid eversion motion occurred faster for shod locomotion compared to barefoot.

Consistent with the findings of Zhang et al. (2013) the barefoot condition yields a shorter stance duration in comparison to walking with conventional sneakers. The findings of Shroyer and Weimar (2010) revealed that compared with sneakers, flip‐flops resulted in a shorter stride length and a shorter stance time. Our findings that stance duration is shorter barefoot than shod on the natural substrate, and that stride duration is shorter barefoot than shod on an artificial substrate, is consistent with the hypothesis that the more minimal the shoe, the shorter the strides and stance.

In addition, the barefoot condition shows a smaller degree of plantarflexion or a larger ankle angle dorsiflexion at toe‐off on both substrates. This is consistent with the findings of Shroyer and Weimar (2010) who revealed that compared with sneakers, flip‐flops resulted in a larger ankle angle/dorsiflexion at the beginning of the double support phase. Keenan (2011) identified potentially clinically relevant changes in joint moments that occur with the shod condition. The most likely causal factor was the increased stride length and its associated changes in ground reaction forces.

The study of Chard et al. (2013) shows that flip flops resulted in increased ankle dorsiflexion during contact both for walking and jogging. The increased ankle dorsiflexion during the contact phase while walking with flip flops, has been suggested to be a mechanism to retain the footwear (Chard et al., 2013). A major difference between the flip flop and the Kolhapuri footwear is the presence of an instep strap which holds the foot close to the outsole in the case of the Kolhapuri footwear. In oher words the compensation that exists while wearing flip flops is not necesssary in case of the Kolhapuri footwear. The wearing of Kolhapuri footwear does not show a significant difference on the angle of ankle dorsiflexion during contact.

Our quantitative analysis shows that the ankle is more plantarflexed at initial impact when walking on the artificial substrate (Figure 5c), regardless of whether the person is shod or unshod. This can be related to a more pronounced heel strike and corresponds with the findings of De Wit, De Clercq, and Aerts (2000) who studied heel strike during running. On the one hand ankle kinematics shows adjustments in foot strike on different substrates (as in running, Ferris, Louie, & Farley, 1998). We also note that the natural soil our subjects walked on is hard and comparable to a layer of buffalo leather (for details see (Nagel, Fernholz, Kibele, & Rosenbaum, 2008)). On the other hand, literature reports that leg stiffness adjustments are accompanied by kinematic and kinetic adjustments. Runners quickly adjust their leg stiffness on their first step when they encounter a new surface such as the transition from a soft to hard surface, which allows them to maintain similar running mechanisms on different surfaces (Ferris, Liang, & Farley, 1999; Kerdok, Biewener, McMahon, Weyand, & Herr, 2002). Hatala, Dingwall, Wunderlich, and Richmond (2013a) mentioned that more compliant surfaces would likely result in lower impact peaks— if attenuation of impact forces is important for the selection of foot strike patterns, then runners may make smaller adjustment to their strike patterns on more compliant substrates

Tillman, Fiolkowski, Bauer, and Reisinger (2002) found no significant differences in shoe reaction forces between four surfaces when running at the same speed (Tillman et al., 2002). It should be stressed that although our data correspond with the literature, we did not control speed and thus speed alone might entirely or partly explain the differences between substrates in our study. Walking speed is known to influence most variables we studied.

Voloshin (2000) studied the effect of walking speed on impact acceleration using an accelerometer mounted onto the tibial tuberosity (therefore his absolute acceleration magnitudes are not directly comparable to ours). Entering the speeds we measured on the artificial and natural substrates into his equation suggests that speed alone would lead to an increase in impact acceleration of 9.6% for the barefoot, and 12.1% for the shod condition. We have measured and increase of 24.7% and 27.7% respectively and therefore we suggest that our impact differences between substrates can be partly, but not fully, explained as an effect of speed alone.

Winter (1991) and Rosenbaum, Hautmann, Gold, and Claes (1994) found relatively small differences for ankle inversion/eversion range over a range of speeds much larger than that between our average speeds on the two substrates. Therefore our general lack of significant differences between conditions in terms of ankle kinematics (Figure 6) is not surprising.

It has been long established that spatiotemporal gait variables are affected by walking speed. Using the step frequency/speed equation in Hediyeh, Sayed, and Zaki (2015), our speed difference would explain a decrease in step duration of 6.8% (barefoot) and 8.8% (shod). We have measured much smaller reductions of approximately 2% where significant. We therefore suggest that the effect of speed on step frequency between substrates is smaller than expected if it were due to speed alone.

Murray, Mollinger, Gardner, and Sepic (1984) showed an almost linear increase, with a slope of approximately 1, of total muscle activity with walking speed for both the pre‐tibial and the calf muscles. Therefore we should expect an increase in integrated sEMG of approximately 7.1% (barefoot) to 9.2% (shod) due to speed differences between substrates. Due to the high variation in our sEMG data we should be very careful to draw conclusions, but we do generally observe a trend for higher integrated sEMG values (on average 6%) on the natural substrate compared to the artificial substrate. Therefore we suggest that potential differences might be due to the effect of speed alone.

Kung, Fink, Hume, and Shultz (2015), using conventional footwear in children, found an increase in the dorsiflexor impulse throughout the stance phase during shod walking, compared to barefoot walking. It is possible that the differences between our conditions were too small and/or variable to show such differences.

Overall, the current study suggests that differences between footwear conditions are subtle and we conclude that walking in Kolhapuri footwear is very similar to barefoot walking. This type of indigenous footwear can be seen to “mimic” barefoot gait to a large extent, whilst offering protection, and might therefore be considered “minimal”. Avenues for future research include a comparison of indigenous footwear with modern, western “minimal footwear”, and an analysis of plantar pressures, where a comparison can be made with literature data for barefoot walking (Bennett and Duplock, 1993; Blanc, Balmer, Landis, & Vingerhoets, 1999; Bryant et al., 2000; Hennig & Rosenbaum, 1991; Hennig et al., 1994) and for barefoot jogging (De Cock, De Clercq, Willems, & Witvrouw, 2005).
